# Supplementary material for: R2R - software to speed the depiction of aesthetic consensus RNA secondary structures
Source: BMC Bioinformatics. 2011 Jan 4;12:3. doi: 10.1186/1471-2105-12-3 (PMC3023696; doi:10.1186/1471-2105-12-3)

SAM-IV

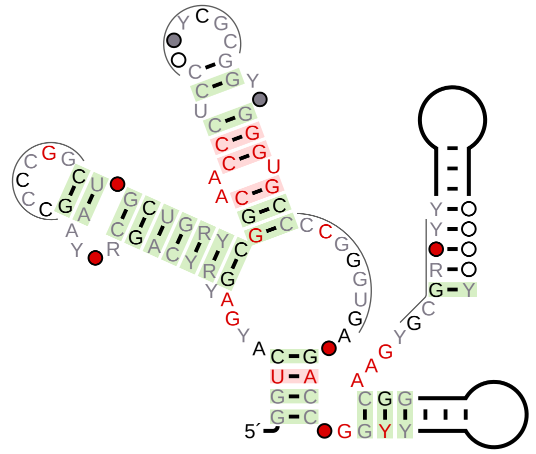

SAM-IV NC_003888.3/2308784-2308334

Shrinking nucs & bonds using
nucShrinkWithCircleNuc and pairBondScaleWithOneSeq
because circle_nuc or #=GR ... CLEAVAGE was used
Set these vars to 1 using SetDrawingParam
to disable. See note1 in manual.

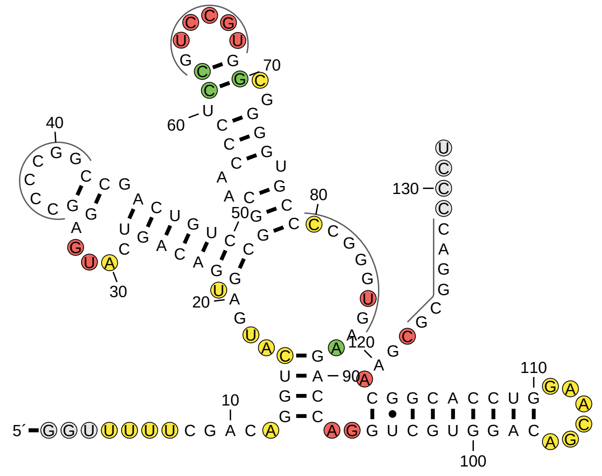

SAM-IV NC_003888.3/2308784-2308334 multistem=original

Shrinking nucs & bonds using
nucShrinkWithCircleNuc and pairBondScaleWithOneSeq
because circle_nuc or #=GR ... CLEAVAGE was used
Set these vars to 1 using SetDrawingParam
to disable. See note1 in manual.

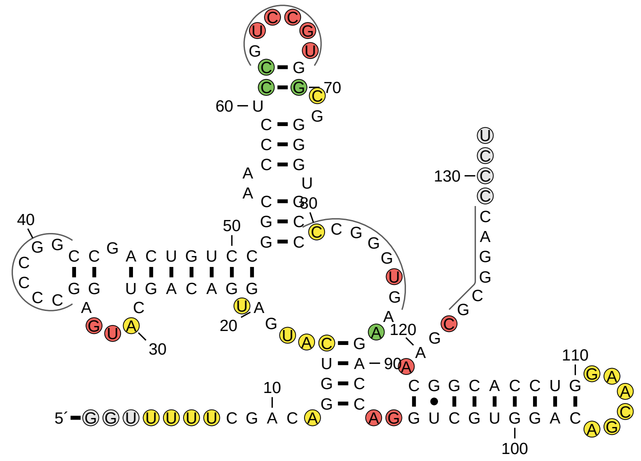

SAM-IV NC_003888.3/2308784-2308334 multistem=solver internalloop=solver2

Shrinking nucs & bonds using
nucShrinkWithCircleNuc and pairBondScaleWithOneSeq
because circle_nuc or #=GR ... CLEAVAGE was used
Set these vars to 1 using SetDrawingParam
to disable. See note1 in manual.

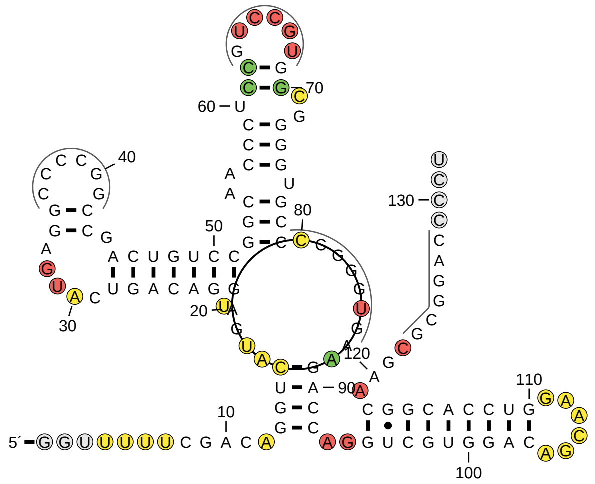

SAM-IV NC_003888.3/2308784-2308334 pseudoknot=1

Shrinking nucs & bonds using
nucShrinkWithCircleNuc and pairBondScaleWithOneSeq
because circle_nuc or #=GR ... CLEAVAGE was used
Set these vars to 1 using SetDrawingParam
to disable. See note1 in manual.

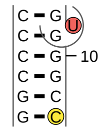

SAM-IV NC_003888.3/2308784-2308334 pseudoknot=2

Shrinking nucs & bonds using
nucShrinkWithCircleNuc and pairBondScaleWithOneSeq
because circle_nuc or #=GR ... CLEAVAGE was used
Set these vars to 1 using SetDrawingParam
to disable. See note1 in manual.

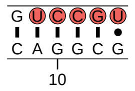

SAM-IV Weinberg2008-Fig1=1 multistem=original

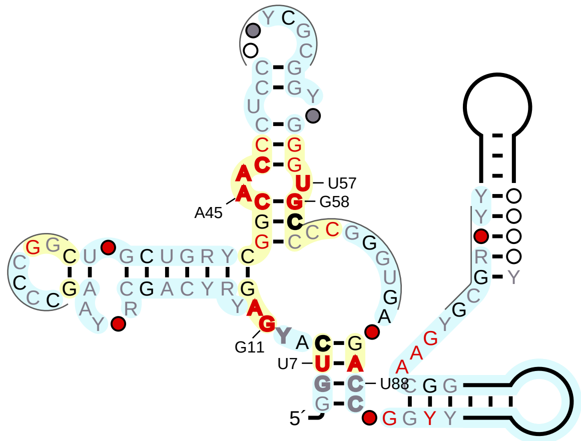

SAM-IV multistem=bulgey

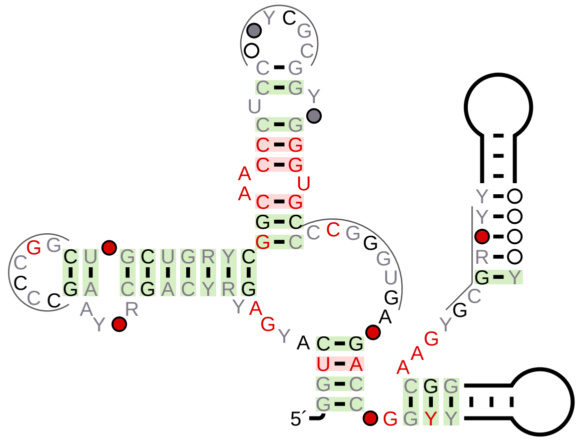

SAM-IV multistem=original

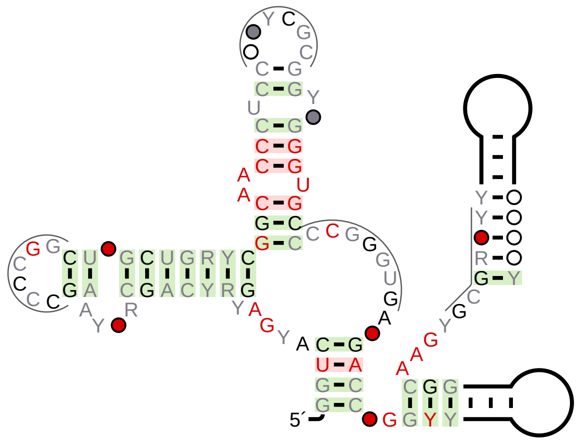

SAM-IV multistem=original internalloop=solver

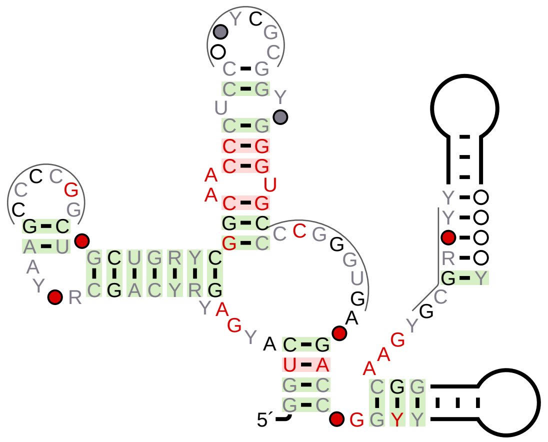

SAM-IV multistem=original internalloop=solver2

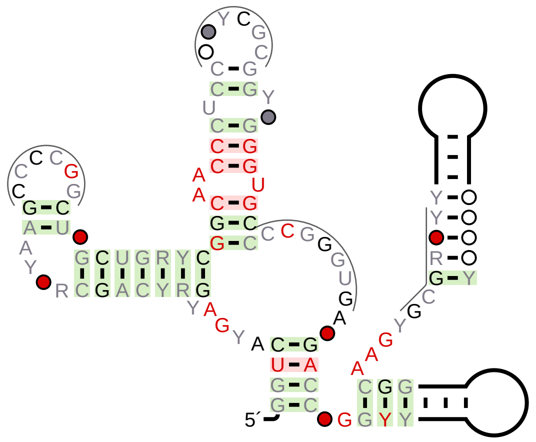

SAM-IV multistem=original internalloop=solver3

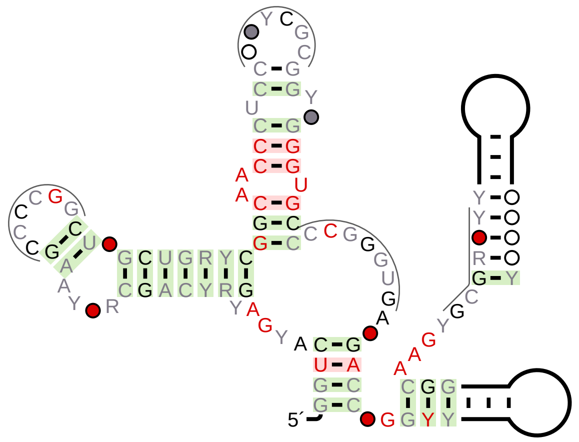

SAM-IV multistem=original internalloop=turn

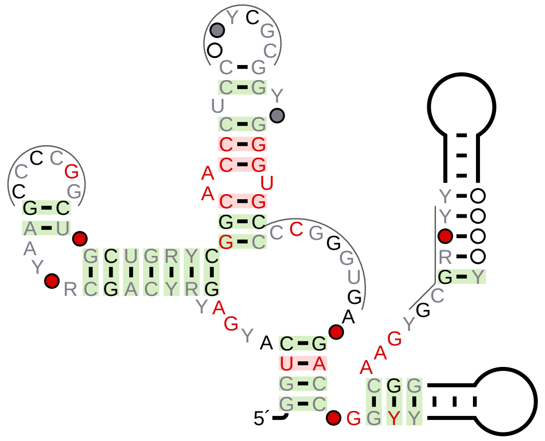

SAM-IV multistem=solver

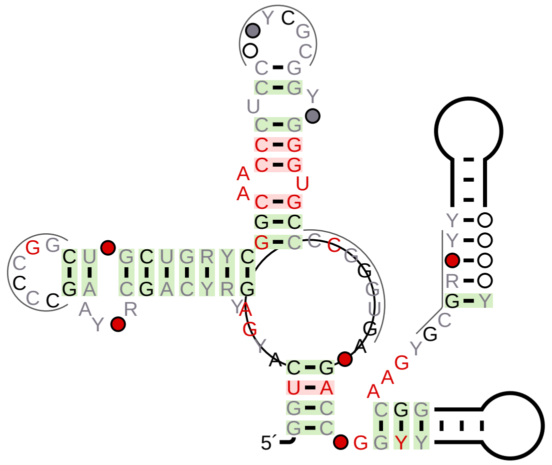

SAM-IV multistem=solver2

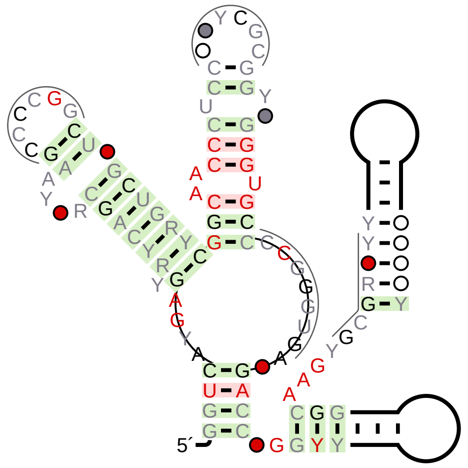

SAM-IV pseudoknot=1

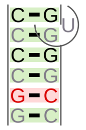

SAM-IV pseudoknot=2

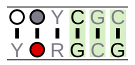

SAM-IV skeleton-with-bp

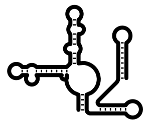

Supplement: Additional file 1 — Source code and example input and output files. C++ and Perl source code is provided. Installation and usage instructions are given in the user manual (Additional file 2), which is also a part of this archive. Example input files for R2R are included in the "demo" subdirectory of this archive, and R2R's raw output on these examples in PDF and SVG format is supplied in the "demo/output-pdf" and "demo/output-svg" subdirectories. Files can be retrieved from the tgz archive file using programs such as WinZip (Windows), StuffIt Expander (Mac) or the tar/gzip commands (UNIX). [file 1471-2105-12-3-S1.TGZ › R2R-1.0.1/demo/output-pdf/SAM-IV.pdf]
